# Recurrent left ventricular pseudoaneurysm in a young patient with Behcet’s disease

**DOI:** 10.1093/ehjcr/ytad544

**Published:** 2023-11-06

**Authors:** Aida Soufiani, Hamza Chraibi, Nesma Bendagha

**Affiliations:** Cardiology A Department, Ibn Sina Hospital, Mohammed V University, Bettouga Street, Rabat 10000, Morocco; Cardiology A Department, Ibn Sina Hospital, Mohammed V University, Bettouga Street, Rabat 10000, Morocco; Cardiology A Department, Ibn Sina Hospital, Mohammed V University, Bettouga Street, Rabat 10000, Morocco

## Case description

A 24-year-old Moroccan patient was admitted to the cardiology department. He had a history of Behcet’s disease (BD) complicated by a large left ventricular pseudoaneurysm for which, 9 years before, he underwent surgical repair with patch closure with favourable post-operative results. He presented with acute heart failure; clinical symptoms included Class IV New York Heart Association dyspnoea alongside bilateral leg oedema. On chest auscultation, bilateral crackles were heard. The rest of the physical examination found specific signs of BD such as oral aphtosis and inflammatory oligoarthritis.

**Figure 1:**
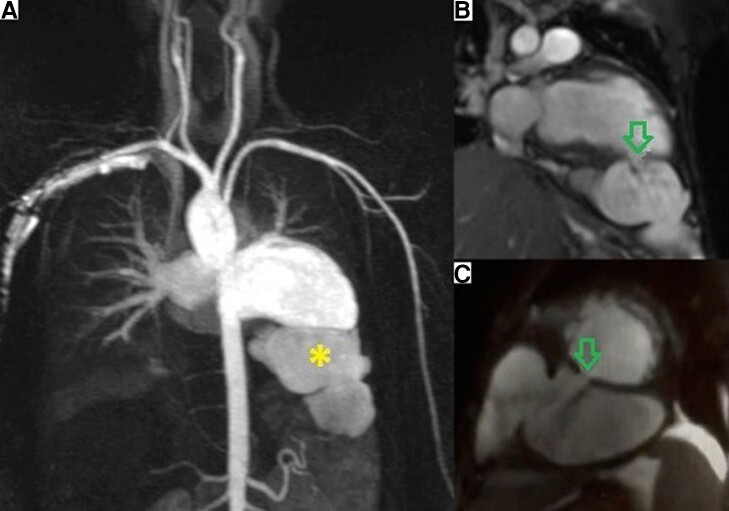
Cardiac magnetic resonance: (*A*) 3D angiography, (*B*) two-chamber view cine sequences, (*C*) short-axis view cine sequences. Star: giant bi-lobed left ventricular pseudoaneurysm. Arrow: rupture of the left ventricular inferior wall.

Cardiac magnetic resonance imaging revealed hypokinetic dilated cardiomyopathy with severe systolic dysfunction. The left ventricular ejection fraction was 20%. There was a rupture of the left ventricle’s inferior wall due to patch dehiscence creating a giant 85 × 52 mm bi-lobed pseudoaneurysm (*Figure 1*, Supplementary material online). Initially, the patient’s clinical state was stabilized using oxygenation therapy as well as loop diuretics. The decision, in accordance with the patient’s wishes, was optimal heart failure medical therapy. He was discharged after an uneventful hospital course.

Behcet’s disease is a systemic vasculitis affecting young adults with mostly cutaneous and articular manifestations. The diagnosis and classification of BD should be made using the 2014 International Criteria for Behcet’s Disease.^1^ Cardiac involvement is present in ∼6% of patients according to the results of a large Tunisian cohort.^2^ Left ventricular pseudoaneurysm is one among many reported complications,^3^ and it is explained by myocardial fragility caused by coronary ischaemia. Cardiovascular surgery in the active inflammatory stage often results in severe post-operative complications such as patch dehiscence. Immunosuppressive therapy and colchicine may help manage such events.^4^ Careful follow-up is required, even for patients with successful initial patch repair. This is, to our knowledge, the first case of recurrent left ventricular pseudoaneurysm associated with BD. This disease should always be suspected in the aetiological workup of pseudoaneurysm, especially in young male patients.

## Supplementary Material

ytad544_Supplementary_Data

## Data Availability

Data sharing is not applicable to this report as no datasets were generated or analysed for this case.

## Notes

**Consent:** Informed consent was obtained from the patient in accordance with COPE guidelines.

**Funding:** None declared.
